# Maternally and zygotically provided Cdx2 have novel and critical roles for early development of the mouse embryo

**DOI:** 10.1016/j.ydbio.2010.04.017

**Published:** 2010-08-01

**Authors:** Agnieszka Jedrusik, Alexander W. Bruce, Meng H. Tan, Denise E. Leong, Maria Skamagki, Mylene Yao, Magdalena Zernicka-Goetz

**Affiliations:** a The Gurdon Institute, University of Cambridge, Tennis Court Road, Cambridge, CB2 1QN, UK; b Present address: Department of Molecular Biology, Faculty of Science, University of South Bohemia, Ceske Budejovice, CZ-37005, Czech Republic; c Department of Obstetrics and Gynecology, Stanford University School of Medicine, Stanford, California, USA

**Keywords:** Cdx2, Trophectoderm, Mouse embryo, Polarisation, Cell death, Compaction

## Abstract

Divisions of polarised blastomeres that allocate polar cells to outer and apolar cells to inner positions initiate the first cell fate decision in the mouse embryo. Subsequently, outer cells differentiate into trophectoderm while inner cells retain pluripotency to become inner cell mass (ICM) of the blastocyst. Elimination of zygotic expression of trophectoderm-specific transcription factor *Cdx2* leads to defects in the maintenance of the blastocyst cavity, suggesting that it participates only in the late stage of trophectoderm formation. However, we now find that mouse embryos also have a maternally provided pool of *Cdx2* mRNA*.* Moreover, depletion of both maternal and zygotic *Cdx2* from immediately after fertilization by three independent approaches, dsRNAi, siRNAi and morpholino oligonucleotides, leads to developmental arrest at much earlier stages than expected from elimination of only zygotic Cdx2. This developmental arrest is associated with defects in cell polarisation, reflected by expression and localisation of cell polarity molecules such as Par3 and aPKC and cell compaction at the 8- and 16-cell stages. Cells deprived of Cdx2 show delayed development with increased cell cycle length, irregular cell division and increased incidence of apoptosis. Although some Cdx2-depleted embryos initiate cavitation, the cavity cannot be maintained. Furthermore, expression of trophectoderm-specific genes, *Gata3* and *Eomes*, and also the trophectoderm-specific cytokeratin intermediate filament, recognised by Troma1, are greatly reduced or undetectable. Taken together, our results indicate that Cdx2 participates in two steps leading to trophectoderm specification: appropriate polarisation of blastomeres at the 8- and 16-cell stage and then the maintenance of trophectoderm lineage-specific differentiation.

## Introduction

The separation of the pluripotent ICM from the trophectoderm by the blastocyst stage is the first cell fate decision in the mouse embryo. The ICM provides progenitors for all cells of the future body, while trophectoderm provides an extra-embryonic tissue, which supports embryo development in the uterus and provides signalling sources to pattern the embryo before gastrulation. The formation of these two tissues occurs in two successive stages. First, cells are allocated to either inside and outside positions via so called differentiative, or asymmetric, divisions that occur in two waves, at the 8- to 16-cell and the 16- to 32-cell stages (Graham and Deussen, 1978; Jedrusik et al., 2008; Johnson and Ziomek, 1982; Pedersen et al., 1986). These divisions contribute to the establishment of inside–outside asymmetry as they distribute key factors for trophectoderm formation, such as cell polarity molecules and Cdx2 mRNA, asymmetrically between the daughter cells (Plusa et al., 2005; Thomas et al., 2004; Jedrusik et al., 2008). Second, once cell divisions have generated inside and outside cell populations, molecular mechanisms sensing cell position can influence transcription from the *Cdx2* locus such that its expression is suppressed in the inner cells but enhanced in outer cells. Recent evidence implicates the Hippo signalling pathway in this mechanism (Nishioka et al., 2009; Nishioka et al., 2008; Yagi et al., 2007). The initiation of the asymmetry in distribution of Cdx2 protein appears to be important for down-regulating the expression of *Oct4* and *Nanog* in the outside cells, and ensuring that the ICM and trophectoderm lineages are segregated by the blastocyst stage (Niwa et al., 2005; Strumpf et al., 2005). Thus, it appears that both cell polarity and cell position affect this first cell fate decision.

Although Cdx2 is a key trophectoderm-specific transcription factor, the stage at which it starts to act and the processes it controls still remain unclear. Embryos in which zygotic expression of *Cdx2* was prevented were reported to develop normally until the late blastocyst stage, which led the authors to suggest that *Cdx2* is not involved in the processes essential for initiation of trophectoderm formation, such as cell polarisation or cell allocation, but only much later in maintenance of trophectoderm (Ralston and Rossant, 2008; Strumpf et al., 2005). However, more recent studies opened up a possibility for an earlier role of Cdx2. First, it was found that up-regulation of Cdx2 expression before the 8-cell stage, affects the extent of cell polarisation and cell allocation to inside versus outside positions: more Cdx2 led to more cell polarity, measured by apical localisation of aPKC and to more symmetric divisions that consequently generate more trophectoderm than ICM (Jedrusik et al., 2008). Second, depletion of Cdx2 before the 8-cell stage in just a part of a normally developing embryo, led to the opposite outcome: Cdx2-depleted cells more often divided asymmetrically contributing to the ICM rather than trophectoderm. Thus, modulating *Cdx2* expression by the 8-cell stage led to an earlier phenotype than that described for the zygotic *Cdx2* knockout. Moreover, this early role of Cdx2 seemed consistent with reports that Cdx2 protein is already present by the 8-cell stage, and thus by the time of cell polarisation and compaction (Jedrusik et al., 2008; Ralston and Rossant, 2008). One possible explanation of these different outcomes would be that one study depleted Cdx2 throughout the whole embryo (Ralston and Rossant, 2008; Strumpf et al., 2005), while the other (Jedrusik et al., 2008) generated embryos in which Cdx2-depleted and Cdx2-expressing cells developed side by side, making it possible to follow the precise behaviour and “competition” between these two cell types by time-lapse studies. The alternative explanation of these different outcomes that there might be a maternal pool of *Cdx2* mRNA which would be eliminated in only one of these studies, as it is susceptible to RNAi, but still present in *Cdx2*^*−/−*^ embryos, initially seemed less likely. This is because the paper claiming existence of the maternal Cdx2 in the zygote has been retracted (Roberts et al., 2007). However, whether there is indeed a pool of maternally inherited *Cdx2* mRNA in the early mouse embryo and whether this has any function have never been rigorously tested.

In this study, we show evidence that mouse embryos have maternally provided *Cdx2* mRNA and that this early pool of *Cdx2* is required for normal development at much earlier stages than previously suspected. We find that depletion of maternal and zygotic *Cdx2* from the early zygote stage leads to developmental arrest associated with abnormal cell polarisation and cell compaction at the 8- to 16-cell stage transition. Such embryos also show slower developmental progression measured by an increased cell cycle length, irregular cell divisions and increased incidence of cell death. These results lead us to propose a model in which Cdx2 is involved in both initiating and subsequently committing proper trophectoderm formation.

## Materials and methods

### Embryo collection and culture

In experiments performed in Cambridge, embryos were collected into M2 medium with 4 mg/ml BSA from 4- to 6-week-old F1 (C57Bl6 × CBA) females superovulated with 7.5 IU of pregnant mare's serum gonadotropin (PMSG; Intervet) and 7.5 IU human chorionic gonadotropin (hCG; Intervet) 48 hours later and mated with F1 or H2B-EGFP males (Hadjantonakis and Papaioannou, 2004). Zygotes were released from ampullae of oviducts 20 hours after hCG and cumulus cells were removed by hyaluronidase treatment and pipetting in M2 medium. Embryos were cultured in drops of KSOM with 4 mg/ml BSA under paraffin oil in 5% CO_2_ at 37.5 °C in groups of 10–15 per 20 μl drop. In one experiment assaying the effect of inhibiting zygotic transcription, embryos were cultured in KSOM supplemented α-amanitin (24 µg/ml) from the 4- to 8-cell transition until the early 16-cell stage when they were fixed. Experiments confirming the efficacy of α-amanitin treatment were first performed by treatment of zygotes with α-amanitin (24 µg/ml–20 hours post hCG) and culturing until the late 2-cell stage, before embryos were harvested for real-time PCR (see below).

In experiments performed at Stanford, involving microinjection of antisense morpholino oligonucleotides, all 3- to 5-week-old wild-type F1 (C57BL6xDBA/2) females (Charles River) were superovulated by intraperitoneal injections of 5 IU of PMSG followed by 5 IU of hCG 48 hours later and mated overnight with wild-type males. Zygotes were released from oviducts 17 hours after hCG injection, pooled from 3 to 6 females in M2 media (Chemicon International), followed by immediate cytoplasmic microinjection and culture in Human Tubal Fluid with 10% serum supplement (In-Vitro Fertilization, Inc.) microdrops under mineral oil in 5% CO_2_ at 37 °C and cultured at 8–10 embryos per 20 μl drop.

### *Cdx2* dsRNA and *Cdx2* siRNA microinjection and time-lapse imaging

dsRNA against *Cdx2* was prepared and microinjected as described previously (Jedrusik et al., 2008) at the concentration of 0.7 µg/µl. A *Cdx2*-specific siRNA (GCAGTCCCTAGGAAGCCAAdTdT) and a control oligo (medium GC: Cat. No. 12935-112) were purchased from the pre-designed Invitrogen catalogue and were diluted to 8 µM prior to microinjection, as per manufacturer's instructions. The success of each injection was monitored by co-injecting mRNA for *DsRed* as a control (0.3 µg/µl). Zygotes were injected 20–22 hours after hCG, cultured to the late 2-cell stage and development of individual embryos and of all their cells were followed in 4D by time-lapse microscopy and analysed with SIMI Biocell software as described previously (Bischoff et al., 2008). Fluorescence and DIC Z-stacks were collected for approximately 72 hours, every 15 minutes, on 15 different planes for each time point, from 2-cell to blastocyst stage. Initiation of cell division was defined as the start of cleavage furrow ingression (in DIC images) and metaphase formation (in fluorescence images). In the case of siRNA injected embryos, development was followed in 10 embryos by time-lapse microscopy and 48 were examined manually by periodic examination.

To examine whether depletion of Cdx2 by dsRNA is specific and can therefore be rescued, Cdx2-dsRNA treated embryos were co-injected with a synthetic mRNA for *Cdx2* (50 ng/μl), a concentration previously shown to be non-toxic (Jedrusik et al., 2008). Development of such embryos was assessed alongside control embryos and embryos injected with only *Cdx2*-specific dsRNA using time-lapse microscopy (as described above) or by regular “manual” inspection of embryos. The rescue experiment was performed twice on a total of 21 embryos.

### Immuno-cytochemical staining

Embryos were fixed in 4% PFA for 20 minutes at 37 °C and treated for immuno-fluorescence as previously described (Plusa et al., 2005). Cdx2 was visualised using mouse antibody (mouse monoclonal, BioGenex) at 1:200 in BSA/Tween and AlexaFluor 488-conjugated anti-mouse secondary antibody at 1:500 (Jackson ImmunoResearch Laboratories). For aPKC, rabbit antibody (Santa Cruz) at 1:200 and AlexaFluor 488-conjugated anti-rabbit antibody at 1:200 (Invitrogen) were used. Trophectoderm-specific cytokeratins were recognised with rat Troma1 antibody (1:100, DSHB, Iowa) and AlexaFluor 488-conjugated anti-rat antibody (1:200). To visualise β-catenin, embryos were fixed in 4% PFA with 0.1% Tween 20 and 0.01% Triton X-100 overnight at 4 °C, permeabilised in 0.55% Triton X-100 in PBS for 15 minutes and blocked in 10% foetal bovine serum in PBS for 1 hour. Rabbit anti β-catenin (Invitrogen) at 1:100 and secondary AlexaFluor 488-conjugated anti-rabbit antibody at 1:200 were used. Cleaved caspase 3 was detected using rabbit anti-caspase 3 (cleaved) antibody (1:1000) and AlexaFluor 488-conjugated anti-rabbit antibody at 1:200 (Invitrogen). For Cdx2 and Eomes co-immuno-staining embryos were fixed in 2.5% PFA for 15 minutes at room temperature. Following fixation embryos were washed in PBS and permeabilised for 30 minutes in 0.25% Triton X-100. Prior to antibody incubation, embryos were blocked in 10% foetal bovine serum in 0.01% Triton X-100. Cdx2 was visualised as described above. For Eomes detection, rabbit anti-Eomes antibody (Abcam) at 1:500 was used. Apoptotic cell death was also confirmed by performing a TUNEL assay on Cdx2-depleted and control embryos (Roche). Embryos were fixed in 4% PFA for 15 minutes at room temperature, washed three times in PBS/PVP and permeabilised for 2 minutes in 0.1% Triton X-100 with 0.1% sodium citrate in PBS on ice. Embryos were then washed three times in PBS/PVP and incubated in TUNEL reaction mixture (Roche) for 1 hour at 37 °C in the dark. As positive control, prior to TUNEL reaction, embryos were incubated in micrococcal nuclease reaction (Bio Labs) for 20 minutes at 37 °C and washed three times in PBS/PVP. As negative control embryos were incubated in label solution only (no enzyme) during TUNEL incubation. After antibody incubations and washes, embryos were mounted in DAPI-Vectashield on poly-lysine slides. Cells were imaged on an Olympus upright confocal.

### Whole-mount RNA fluorescence *in situ* hybridisation (RNA FISH)

*FISH* was performed according to Chazaud et al. (2006). To counter-stain nuclei, embryos were treated with 300nM DAPI (Molecular Probes) in PBS. Fluorescence was detected on an LSM510 META laser scanning confocal microscope (Zeiss) with a 40× Plan-Neofluar oil immersion objective. RNA probes for *Cdx2* and *Emx2* (negative control) were generated by the direct *in vitro* transcription of PCR-generated DNA template. Cdx2 antisense probe derived using Cdx2F1/Cdx2R1T7 primer pairs and sense probe using Cdx2F1T7/Cdx2R1 primers (Cdx2F1—TCGCCACCATGTACGTGAGCTACCT; Cdx2R1—TTCAGACCACGGGAGGGGTCACTG; Cdx2F1T7—TAATACGACTCACTATAGGGATGTACGTGAGCTACCTTC; Cdx2R1T7—TAATACGACTCACTATAGGGAGGGGTCACTGGGTGACAG). Antisense probe for Emx2 derived using Emx2F1/Emx2R1T7 primer pairs (Emx2F1—TGAATGATCCTTGTGAGGC; Emx2R1T7—TAATACGACTCACTATAGGGCCTGCTCCCTCATTTCTC).

### Real-time RT-PCR

**Fig. 1:**
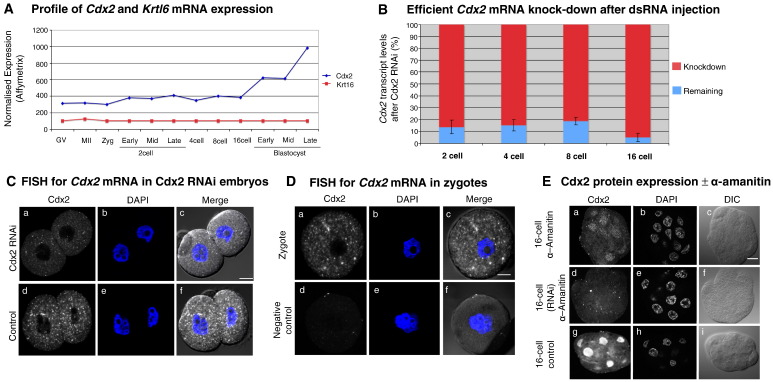
Cdx2 mRNA is present throughout all pre-implantation stages. (A) Developmental profile of *Cdx2* (blue) and *Krt16* (red) mRNA expression from the GV stage oocyte to late blastocyst stage based on previous microarray data (Wang et al., 2004). Note higher *Cdx2* expression relative to basal *Krt16* expression and detectable *Cdx2* mRNA expression in the GV and MII arrested oocyte (MII), zygote and 2-cell stages. (B) Detectable *Cdx2* mRNA, assayed by quantitative RT-PCR, is significantly depleted by the 2-cell stage after microinjection of dsRNA specific for *Cdx2* in the early zygote. This depletion is maintained until the 16-cell stage (development measured against that of control embryos). *Cdx2* mRNA levels at each developmental stage were quantified and normalised to the level of *Actb* transcript for both control and Cdx2-RNAi embryos. The percentage reduction in mRNA expression (and by inference percentage remaining transcript) accounted for by *Cdx2*-specific RNAi was calculated and plotted (errors equal SEM of triplicate measurements). (C) RNA fluorescent *in situ* hybridization (FISH) after Cdx2-RNAi. Representative image of FISH for *Cdx2* mRNA in a 2-cell embryo after *Cdx2*-specific RNAi from the early zygote stage (sub-panels ‘a–c’). Note significantly reduced signal compared to uninjected control embryo image (sub-panels ‘d–f’). DAPI staining was used to stain nuclei. Scale bar 10 μm. (D) RNA FISH assay in the early zygote for *Cdx2* mRNAs. Representative embryo image for *Cdx2*-specific probe (sub-panels ‘a–c’) and negative control *Emx2* (neural gene not expressed during pre-implantation development) probe (sub-panels ‘d–f’) are shown. DAPI staining was used to stain nuclei. Scale bar 10 μm. (E) Immuno-fluorescence staining specific for Cdx2 protein in representative embryos at 16-cell stage (with or without exposure to α-amanitin or *Cdx2*-specific RNAi). DAPI DNA counter-stain and phase images are shown for reference. Note that Cdx2 expression at 16-cell stage is in large part derived from the translation of zygotically derived transcripts (compare sub-panels ‘a’ and ‘g’). However, vestigial staining (sub-panel ‘a’) reflects protein synthesised from transcript present in embryos by the 4-cell stage. This Cdx2 protein is lost if the embryos are pre-treated with *Cdx2*-specific RNAi (sub-panel ‘d’). Control embryo group *n* = 10, α-amanitin alone treated group *n* = 8 and α-amanitin plus *Cdx2* RNAi treated group *n* = 7. Scale bar 10 μm.

Total RNA was prepared from embryos that had been microinjected with dsRNA specific for *Cdx2* transcript (and *DsRed* mRNA to confirm injection) at the early zygote stage and subsequently cultured to the mid 2-, 4-, 8- or 16-cell stage. Similarly, RNA was also prepared from control embryos that had been cultured from the zygote stage after injection with *DsRed* mRNA only. Additionally, mRNA was also prepared from 2-cell embryos cultured with or without α-amanitin from the early zygote stage. Fifty embryos for each condition were transferred to 20 μl of extraction buffer (Arcturus Biosciences; ‘PicoPure RNA isolation kit’) and mixed with 20 μl of 70% ethanol. After following the manufacturer's protocol, total RNA was eluted into 10 μl of water and any contaminating DNA digested by DNaseI treatment (Ambion; ‘DNA-*free*’ kit). All the resulting uncontaminated RNA was then reverse transcribed using oligodT priming in 20-μl reactions (Invitrogen; ‘Superscript III Reverse Transcriptase’). Synthesised cDNA (0.5 μl per reaction) was then used as template in 25-μl real-time reactions (Applied Biosystems: ‘SYBR Green master-mix’) using oligonucleotide primers (final conc. 400nM) specific for either mouse *Actb* (GCTCTTTTCCAGCCTTCCTT and CGGATGTCAACGTCACACTT), *Cdx2* (TCAAGAAGAAGCAGCAGCAG and GCAAGGAGGTCACAGGACTC), *Eomes* (TCAGATTGTCCCTGGAGGTC and CTCTGTTGGGGTGAGAGGAG) *Tead4* (GAGCCCGGAGAACATGATTA and CCAAATGAGCAGACCTTCGT), *Gata3* (CCGAAACCGGAAGATGTCTA and AGATGTGGCTCAGGGATGAC), *Oct4* (GGAAAAGGGACTGAGTAGAGTGTGG and TTGGGCTAGAGAAGGATGTGGTT), *Nanog* (TGCAATGGATGCTGGGATACTC and GGTTGAAGACTAGCAATGGTCTGA), *E-cadherin* (AGACTTTGGTGTGGGTCAGG and CATGCTCAGCGTCTTCTCTG), *aPKC* (AGCCCCAGATCACAGATGAC and TCAAATTCGGACTGGTCGAT), *Par1* (CCCATTGACACCATCAACTCT and TGTGGAACCTCTCCCTGACT), *Par3* (AGCCTTCTGGTCTTTCGTCA and GGGTGTGAGAACAACGTCCT), *Eif1a* (AGGCGCAGAGGTAAAAATGA and ATATGGCACAGCCTCCTCAC) or *Mdm4* (GCGCGAGAGAACAAACAGAT and GGCTCGTCTTCCCATGAATA) transcripts. All transcript levels were normalised against *Actb*, in each condition, using the ∆∆Ct method (Livak and Schmittgen, 2001) and expressed as percentage of total knockdown within a particular stage (assaying *Cdx2* mRNA after RNAi: Fig. 1B), as relative expression fold change (after *Cdx2* RNAi at 16-cell stage: Fig. 4B) or normalised absolute expression versus *Actb* (Fig. 4B′).

### Microinjection of antisense morpholino oligonucleotides

25-nt, antisense morpholino oligonucleotides (MOs) that specifically target the translational start site or 5′UTR were purchased from Gene Tools, LLC. The sequence for these morpholinos are as follows: *Cdx2*-MO1 5'-TGTCCAGAAGGTAGCTCACGTACAT-3'; *Cdx2*-MO2 5'-AGGGACCCAGAGCAGACCTCACCAT-3'; Control-MO 5'-TCCAGGTCCCCCGCATCCCGGATCC-3'. We had previously determined 0.6 mM to be the maximal concentration that would allow normal rates of blastocyst development (data not shown). Hence, unless otherwise specified, 5–10 pl of 0.6 mM of either *Cdx2*-MO1, *Cdx2*-MO2 or Control-MO was injected into the cytoplasm of each zygote on an inverted microscope (Olympus IX70) equipped with hydraulic micromanipulation system (IM300 Microinjector, Narishige, Japan). At least 8–10 embryos were used for each of the conditions, uninjected, control-MO, *Cdx2*-MO1 and *Cdx2*-MO2 in each experiment, which was performed three times, except for *Cdx2*-MO2, which was tested two times.

### Control morpholino oligonucleotides

In each experiment, uninjected embryos and embryos injected with a control morpholino (Control-MO) were tested in parallel with *Cdx2-*MO1*-* and *Cdx2-*MO2-mediated knockdown. The Control-MO was designed to specifically target the human globin gene promoter (Gene-tools, Inc.), which is not present in the mouse genome. We had tested this Control-MO morpholino when establishing our methods and found that its presence did not affect blastocyst developmental rates. Importantly, genes that were previously validated to be differentially-expressed between uninjected and *Oct4*-MO-injected embryos were also confirmed to show no differential expression between un-injected and Control-MO-injected embryos (Foygel et al., 2008).

### Statistical analysis

The mean percentage and standard error of the mean (mean ± SEM) of embryos progressing to, or arresting at, each developmental stage were calculated, and statistical significance was determined by calculating the *p*-value using two-tailed Student's *t*-test.

## Results

### Maternal *Cdx2* mRNA is present in the early mouse embryo

We have previously described genome-wide patterns of mRNA expression throughout the pre-implantation stages of mouse development (Wang et al., 2004). In closely analysing these data, we were surprised to note low, yet significant, expression of the trophectoderm-specific transcription factor *Cdx2* in GV stage and MII arrested oocytes, zygotes and 2-cell stage embryos (Fig. 1A). Although expression of *Cdx2* has been previously reported at these earlier stages (Deb et al., 2006), this paper was later retracted (Roberts et al., 2007), leaving the prevailing view that *Cdx2* mRNA is not expressed until after the activation of the zygotic genome and not earlier than at the 8-cell stage (Jedrusik et al., 2008; Ralston and Rossant, 2008). With this in mind, we decided to independently verify the presence of *Cdx2* mRNA during these earlier stages, using both quantitative RT-PCR and RNA fluorescence in situ hybridisation (FISH) approaches (Figs. 1B–D). Quantitative RT-PCR allowed us to detect *Cdx2* mRNA at the 2-, 4-, 8- and 16-cell stages. Moreover, we found that this *Cdx2* mRNA could be efficiently depleted by the 2-cell stage by injection of dsRNA specific for *Cdx2* into the early zygote as judged by both quantitative RT-PCR (Fig. 1B) and by FISH (Fig. 1C). Together, these multiple lines of experimentation provide evidence that *Cdx2* mRNA must have a maternal origin in mouse embryos, as suggested by the microarray analysis (Fig. 1A), since the major burst of zygotic genome activation (ZGA), and hence zygotic transcription, only occurs at the late 2-cell stage. A FISH assay of early zygotes confirming the presence of *Cdx2* transcripts further supports this interpretation (Fig. 1D).

We could detect the first clear localisation of Cdx2 protein in blastomeres' nuclei at the 8-cell stage. The presence of Cdx2 protein was very heterogeneous at this stage with some blastomeres having clearly much higher Cdx2 levels than others (Supplementary Fig. 1), in support of some previous observations (Yagi et al., 2007; Jedrusik et al., 2008; Ralston and Rossant, 2008), but in contrast to another which described absent or low levels of Cdx2 at the 8-cell stages becoming up-regulated in all 8-cell blastomeres (Dietrich and Hiiragi, 2007). It is possible that Cdx2 protein is present at an even earlier developmental stage, but the sensitivity of available antibodies against Cdx2 do not allow, in our hands, its robust and reproducible detection before the 8-cell stage. From the 16-cell stage onwards, Cdx2 levels increased consistently with the increase in *Cdx2* transcripts from this time (Fig. 1A). In order to distinguish between protein made from the early pool of *Cdx2* mRNA from that made after robust up-regulation of zygotic *Cdx2* at the 16-cell stage (Fig. 1A), we used α-amanitin to block transcription from the 4- to 8-cell stage transition until the early 16-cell stage and assessed Cdx2 protein levels by immuno-fluorescence. After confirming the efficacy of α-amanitin treatment (Supplementary Fig. 2), we found that inhibition of transcription prevented the increase in Cdx2 protein at the 16-cell stage, in comparison to control embryos, although low levels of Cdx2 were clearly detectable in cell nuclei (Fig. 1E), indicating that this protein was the result of translation of transcripts already present by the 4-cell stage. Although we cannot eliminate a possibility that these transcripts may be the products of early zygotic transcription, the fact that *Cdx2* mRNA levels change very little from the zygote to 16 cell stages (Fig. 1A) and that after the depletion of this early pool of *Cdx2* transcripts by RNAi, the presence of this Cdx2 protein was lost (Fig. 1E), provides further supporting evidence of the existence of maternal *Cdx2* mRNA that become translated as development progresses.

### Depletion of maternal and zygotic Cdx2 from the zygote stage affects development before the blastocyst stage

This unexpected detection of an early pool of *Cdx2* mRNA raised the question of whether it has any developmental function. To address this, we first used an RNAi approach that has been shown previously to be highly effective in assessing gene expression in oocytes and pre-implantation mouse embryos (Wianny and Zernicka-Goetz, 2000; Svoboda et al., 2000), as this would allow us to eliminate both the detected maternal and subsequent zygotic transcripts at the same time. This approach therefore would offer a different perspective from the *Cdx2*^*−*^^*/*^^*−*^ knockout study (Strumpf et al., 2005), where any maternal contribution of Cdx2 from the heterozygous *Cdx2*^*+/*^^*−*^ mother would persist and so mask or delay the onset of phenotype in homozygous *Cdx2*^*−*^^*/*^^*−*^ embryos.

**Fig. 2:**
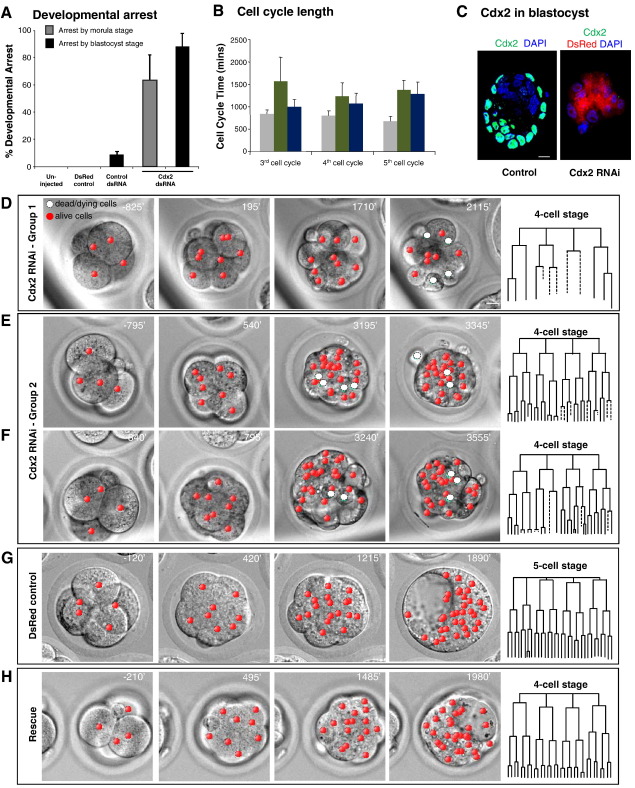
Cdx2 depletion from immediately after fertilization results in pre-implantation arrest. (A) Average frequency of developmental arrest in Cdx2-RNAi embryos (*DsRed* mRNA co-injected as lineage marker; *n* = 18 embryos) before morula (grey bar) and before blastocyst stages (black bar) and three control groups: non-injected embryos (*n* = 15 embryos), embryos injected with mRNA for *DsRed* only (*n* = 20 embryos), embryos injected with dsRNA for gene not involved in lineage specification (*n* = 37 embryos). Significantly lower numbers of Cdx2-depleted embryos showed successful development, compared to near-100% successful development in controls (*t*-test, *p* < 0.05 when comparing knockdown group to any control group). (B) Average cell cycle length (minutes) for third, fourth and fifth cell cycles in control embryos injected at the zygote stage with *DsRed* mRNA alone (grey bars; *n* = 20) or embryos injected *DsRed* mRNA and *Cdx2* dsRNA and exhibiting most severe (group 1—green bars; *n* = 9) or milder phenotype (group 2—blue bars; *n* = 9). Errors equal standard deviation from mean. (C) Sections through fixed and immuno-cytochemically stained embryos at a stage equivalent to the blastocyst stage. Non-manipulated embryos exhibit normal robust Cdx2 staining in the trophectoderm and no staining in the ICM (Control) whereas Cdx2 protein is absent in equivalent embryos injected with dsRNA for *Cdx2* at the zygote stage (Cdx2 RNAi). These embryos exhibit DsRed fluorescence due to co-injection of *DsRed* mRNA as a lineage tracer. Nuclei stained with DAPI (blue); scale bar 10 μm. (D–F) Cdx2-depleted embryos were followed by time-lapse microscopy to a stage equivalent to blastocyst under control conditions; time of images in minutes relative to 8-cell stage entry; scale bar 10 μm. Development of individual cells in each embryo was followed using SIMI Biocell software. Two distinct groups of embryos were distinguished: embryos blocking their development by the 8- to 16-cell stages (D) and embryos arresting between 16-cell and blastocyst stage (E, F). Note lack of compaction in the first group (D) and defects in cavity formation in the second group (E, F). Merged 3D representations and DIC images are shown. The centres of the nuclei of individual cells are marked in red. From fourth cleavage onwards, cell death was observed: centres of nuclei of cells that just died or are about to die are marked white. (G) Representative example of control *DsRed* mRNA injected embryo that developed to the blastocyst stage. (H) Representative example a ‘rescued’ embryo that had been co-injected with *Cdx2*-specific dsRNA and *Cdx2* mRNA (plus *DsRed* mRNA as a lineage marker) that developed to the blastocyst stage in contrast to those injected with *Cdx2*-specific dsRNA alone. In this group 80% (*n* = 21) of embryos exhibited ‘rescued’ development to the blastocyst stage. Schematic representations of lineage trees for all embryos shown in panels D, E, F, G and H are shown on the right. Dashed lines represent cells that died.

To assess the function of this early pool of Cdx2, we down-regulated its expression by injecting early zygotes, immediately after fertilization, with dsRNA for *Cdx2*, which we previously shown to specifically eliminates *Cdx2* mRNA in the mouse embryo (Jedrusik et al., 2008). We confirmed that this treatment led to down-regulation of *Cdx2* mRNA by the 2-cell stage (Figs. 1B, C) and that Cdx2 protein remained depleted until the blastocyst stage (Fig. 2C). In order to characterise in detail developmental progression of Cdx2-depleted along side control embryos, we filmed them from the late 2-cell to the blastocyst stage. By taking a series of 15 optical sections through the embryo at each time point allowed us to follow the timing and orientation of all cell divisions, cell positions and behaviour of all individual cells for 72 hours, thus until their reach the blastocyst stage. With the help of the Simi Biocell software (Schnabel et al., 1997), we generated lineage trees for all experimental and control embryos (Figs. 2D–H).

**Fig. 3:**
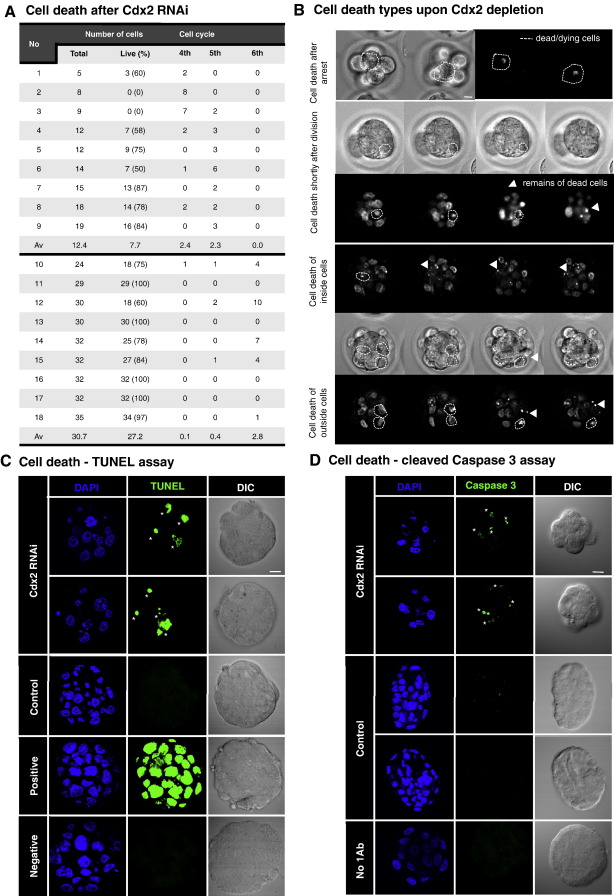
Cdx2 depletion from immediately after fertilization leads to increased cell mortality. (A) Tabularised summary of data on the frequency of cell death observed in Cdx2 depleted embryos (see text for details). (B) Examples of cell death in response to *Cdx2* depletion. Cells could die after blocking their development for a long time at certain developmental stage (top panel first row—cell died after being arrested at the fourth cleavage for nearly 54 hours) or sooner after division (top panel lower two rows—cell died 2.5 hours after division). In embryos that developed beyond 16-cell stage, cell death was observed in both inside and outside cells (bottom panel—top row shows death of inside cell, and bottom row—death of two outside cells in the same embryo). DIC and corresponding GFP (visualising nuclei morphology) images are shown. White dashed lines indicates localisation of cell undergoing cell death on both DIC and GFP images and white arrowheads the remains of dead cells; scale bar 10 μm. (C-D) Characterization of cell death associated with Cdx2 depletion from the zygote stage. (C) Control and Cdx2-depleted embryos were cultured to a stage equivalent to the early blastocyst stage (judged by control embryos) and then subjected to a TUNEL assay. In Cdx2-depleted embryos, stained nuclei indicate apoptotic cell death (*) that is absent from control un-manipulated embryos or negative staining controls. A positive control embryo that had been incubated in micrococcal nuclease prior to TUNEL assay staining shows staining in all nuclei, confirming apoptotic cell death. (D) Control and Cdx2-depleted embryos were cultured to a stage equivalent to the blastocyst stage (judged by control embryos), fixed and immuno-stained for cleaved caspase 3 (a marker of activated apoptotic pathway). Cleaved caspase 3 immuno-reactivity was only observed in the nuclei of Cdx2-depleted embryos (*) and not in control embryos. Note also the difference in DAPI staining between the two groups with Cdx2-depleted embryos exhibiting nuclear fragmentation. A staining control embryo in which primary anti-cleaved caspase 3 antibody was omitted is shown for reference; scale bar 10 μm.

We found that depletion of both maternal and zygotic Cdx2 led to developmental arrest of 88.6 ± 10.3% (*n* = 18) embryos compared to 0% of developmental arrest for the two control groups of embryos, either non-injected (*n* = 15) or injected with mRNA for *DsRed* only (*n* = 20) and 8.6 ± 2.4% injected with dsRNA for a control gene (mean ± standard error of the mean, SEM) (Fig. 2A). This developmental arrest was significantly higher than those of all three control groups (*t*-test, *p* < 0.05). Importantly, we observed that 63.2 ± 18.8% of such *Cdx2*-depleted embryos arrested prior to blastocyst cavitation. The live imaging approach allowed us to distinguish two separate groups of embryos based on onset of developmental defects when compared to control embryos (Figs. 2D–G). The first group, which comprised half of all embryos, consisted of those that arrested already at 8- to 16-cell transition (Figs. 2D and 3A; Supplementary Movie 1). The majority of embryos in this group (67%; *n* = 9) neither underwent compaction nor initiated cavitation, in stark contrast to embryos in which only the zygotic *Cdx2* was eliminated, in which the first defects were reported much later at the blastocyst stage (Ralston and Rossant, 2008; Strumpf et al., 2005). Only in two embryos was cavity formation initiated, but in both of these cases the cavity collapsed soon after its formation. The second group of embryos also arrested, but at slightly later stages (Figs. 2E, F and 3A). We found that although these embryos were able to progress beyond the 16-cell stage and often initiate compaction (89%; 8/9) and cavitation (78%, 7/9), these processes were much delayed. Moreover, embryos in this group also showed morphological abnormalities such as a pre-compaction appearance and increased incidence of cell death (Fig. 3A, see also later). Time-lapse imaging of these embryos revealed that their cavities collapsed (Supplementary Movies 2 and 3). Control embryos showed normal development to the blastocyst stage (Fig. 2G).

We also analysed the cell cycle progression upon Cdx2 down-regulation. This revealed that Cdx2-depleted embryos displayed unusually increased cell cycle lengths compared to control embryos. In the first group of embryos, this started to be evident already at the third cell cycle, the stage immediately preceding that in which most blastomeres arrested (Fig. 2B). In the second group, the increased cell cycle lengths were less pronounced although the duration of the fifth cell cycle was particularly increased prior to developmental arrest (Fig. 2B).

Although it has been previously demonstrated that microinjection of dsRNA into the oocyte or zygote has no adverse effect on development, unless the injected dsRNA targets transcripts with integral roles (Svoboda et al., 2000; Wianny and Zernicka-Goetz, 2000), we carried out three control sets of experiments to address whether the above described phenotypic effects were specific to *Cdx2* depletion. In the first set of control experiments, we microinjected embryos with control dsRNA or with *DsRed* mRNA alone to control for an injection procedure and monitor their developmental progression by time-lapse observations. We found both group of control embryos did not have any defects in their developmental progression or cell cycle length (Figs. 2A, B and G). In the second set of control experiments, we microinjected zygotes with *Cdx2*-specific dsRNA but also with a synthetic *Cdx2* mRNA, at a concentration previously shown to be non-toxic (Jedrusik et al., 2008). Time-lapse observations revealed that 80% (*n* = 21; two independent experiments) of such Cdx2-RNAi embryos were completely rescued by injecting them with synthetic *Cdx2* mRNA and reached the cavitated blastocyst stage in contrast to embryos injected in parallel with *Cdx2*-specific dsRNA alone (Fig. 2H and Supplementary Movie 5), providing evidence that the Cdx2-RNAi was specific for *Cdx2* transcripts. Finally, in the third set of control experiments, we followed the effects of down-regulating Cdx2 by an independent construct, Cdx2-specific siRNA purchased from the Invitrogen siRNA catalogue (Supplementary Fig. 3A; Supplementary Movie 4). We found that 69% (*n* = 58) of the Cdx2 siRNA-treated embryos arrested at the multicellular stage without initiating cavitation, similarly to group 1 of dsRNA Cdx2-depleted embryos described above. Although the remaining embryos developed further and could form ‘blastocyst-like’ structures, their development was delayed and showed similar defects to those observed in the group 2 of dsRNA Cdx2-depleted embryos. We confirmed that microinjection of Cdx2 siRNA led to down-regulation of *Cdx2* by Cdx2-specific immuno-staining, which revealed that the most severely affected embryos had little or no detectable Cdx2 protein, while the more mildly affected embryos had much lower Cdx2 protein levels when compared to controls (Supplementary Fig. 3B). When negative control siRNA were injected at the same developmental stage, 83.3% (*n* = 18) of embryos developed normally to the blastocyst stage. Accordingly, the siRNA phenotype is in strong accord with that obtained using a long dsRNA RNAi-based approach.

Taken together, our results indicate that depletion of an early pool of *Cdx2* has a severe effect upon normal development. Such defects are first clearly manifested at the 8- to 16-cell transition, indicative of a hitherto unrealized early functional role of Cdx2 during pre-implantation development.

### Depletion of maternal and zygotic *Cdx2* from the zygote stage affects cell survival and cell allocation

To gain further insight into the underlying reasons for such severe developmental defects upon maternal and zygotic Cdx2 depletion, we first analysed the behaviour of every single individual cell within the embryo as development progressed. This revealed unexpectedly high frequency of cell death in 78% (*n* = 18) of *Cdx2*-depleted embryos (Figs. 3A, B). On average, 4 cells (4.1 ± 3.5) per embryo died, although in some embryos all cells died (Fig. 3A). We used two assays to characterise this cell death further: the TUNEL assay and immuno-reactivity for cleaved caspase 3, both of which indicated an apoptotic mechanism (Figs. 3C, D). In those embryos that had progressed beyond the 8-cell stage and so had both inside and outside cells, similar numbers of inner as outer cells died (on average 1.6 ± 1.8 inside versus 1.9 ± 3.0 outside) (Fig. 3B; Supplementary Table 1). Thus, cell death in *Cdx2*-depleted embryos did not discriminate between these compartments. This was an unexpected result as cell death is normally observed not earlier than at the blastocyst stage and was reported to be confined to the ICM (Copp, 1978). Moreover, elimination of Cdx2 by the same RNAi approach, but at a later developmental stage, in our previous studies did not result in cell death (Jedrusik et al., 2008).

The analysis of the precise spatial allocations of cells and their numbers in those Cdx2-depleted embryos that progressed beyond 16-cell stage, revealed that the contribution of cells to the inside part of the embryo was slightly greater than in control embryos. On average, 41.3% of the total number of cells were in inside positions compared to 32.4% in the control embryos, injected with *DsRed* mRNA alone (Supplementary Table 1). This increased inner contribution after Cdx2-depletion is in agreement with previous observations that cells with lower Cdx2 levels tend to contribute more to the ICM (Jedrusik et al., 2008). Taken together, these results provide evidence that correct pre-implantation development and spatial allocation of cells to outside positions involve *Cdx2* expression and suggest that Cdx2 function is specific to developmental stage.

### Depletion of early pool of Cdx2 affects cell polarity and blastocyst cavitation

**Fig. 4:**
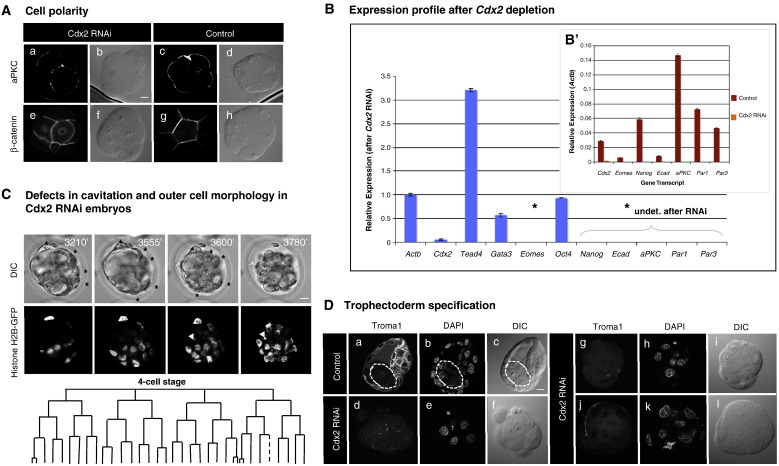
Cdx2 depletion from the zygote stage affects cell polarisation, outer cell morphology and expression of trophectoderm-marker genes. (A) To observe effect on cell polarisation, Cdx2-depleted zygotes were fixed at the 8-cell stage and immuno-stained for aPKC or β-catenin. Comparison of expression level of these factors in Cdx2-depleted (left) and a control embryo (right) are shown. For each factor, images were taken using the same laser settings and phase images are also shown. Note decreased expression of apically localised aPKC protein and increased β-catenin in the nucleus, in the Cdx2-depleted embryos. Scale bar 10 μm. (B) Quantitative real-time PCR analysis of trophectoderm (*Cdx2*, *Tead4, Gata3* and *Eomes*), pluripotency-related (*Oct4* and *Nanog*) and polarity-related (*Ecad, aPKC*, *Par1* and *Par3*) gene mRNA levels at the mid 16-cell stage after *Cdx2*-specific RNAi from zygote stage (normalised to *Actb* levels). Expression levels are shown as fold change, comparing control embryos (injected with *DsRed* mRNA alone) with *Cdx2*-depleted embryos (injected with *Cdx2*-specific dsRNA and *DsRed* mRNA). Errors equal SEM of triplicates. Highlighted transcripts (*) denote those whose expression was reduced to undetectable levels after *Cdx2* RNAi. Accordingly, the mRNA expression levels of these genes in control embryos (relative to that of *Actb*) are shown in the B′ panel to confirm the primers used. (C) Even in Cdx2-depleted embryos that developed beyond 16-cell stage, outer cells morphology was changed and cavitation affected. Representative time-lapse DIC (upper panels) and GFP images (middle panels) of an embryo undergoing cavitation are shown; scale bar 10 μm. Lineage tree for the same embryo generated using SIMI Biocell software (lower panel). White arrowhead on the GFP images and dashed branch of the lineage tree indicate cell death; time of images in minutes relative to 8-cell stage entry is shown. Black stars on DIC images highlight outer cells with abnormal (rounded) morphology prior to cavity collapse (last image of the sequence). (D) Expression of trophectoderm-specific cytokeratins recognised by Troma1 antibody is dramatically reduced after Cdx2 depletion. Immuno-fluorescence staining for Troma1 antigen in representative control blastocysts (panels ‘a–c’—dashed line outlines ICM) and Cdx2-depleted embryos, at a blastocyst equivalent stage (panels ‘d–l’) are shown. DNA DAPI counter-stain and phase images are shown for reference; scale bar 10 µM.

As described above, continuous monitoring of embryogenesis revealed that developmental phenotypes associated with Cdx2 depletion from immediately after fertilization become initiated much earlier than previously suspected and relate to problems with cell division, cell compaction and allocation. This led us to examine whether the depletion of this early pool of *Cdx2*, might affect apical-basal polarisation of blastomeres that is initiated at the 8-cell stage (Johnson and Ziomek, 1982). To this end, we examined the expression and spatial localisation of a number of known polarity markers at both the mRNA and protein levels at the 8-cell and 16-cell stages (Fig. 4). We found that 80% of embryos (*n* = 10) deprived of Cdx2 showed expression of aPKC protein at the 8-cell stage, but its apical localisation was clearly decreased, in comparison to control embryos (Fig. 4A). Similar mis-localisation and down-regulation of aPKC protein was found in embryos targeted with *Cdx2*-specific siRNA (Supplementary Fig. 3C). By the mid 16-cell stage, the *aPKC* mRNA expression in *Cdx2*-depleted embryos was undetectable in comparison to control embryos (Figs. 4B, B′). In addition, other polarity marker gene mRNAs, such as *Par1*, *Par3* and *E-cadherin*, were similarly undetectable after Cdx2 depletion at this stage (Figs. 4B, B′), indicative of substantial defects in cell polarisation. We also observed increased β-catenin protein levels in the nuclei of Cdx2-depleted 8-cell embryos (8/9 embryos) consistent with a defect in the β-catenin localisation mechanism (Fig. 4A), possibly related to the reduced *E-cadherin* mRNA levels which were observed by the 16-cell stage. The consequences of this disrupted cell polarisation were apparent at the time of cavitation, even in embryos with seemingly unaffected development (as judged by the lineage tree generated with SIMI Biocell software which seemed relatively normal; Fig. 4C). Thus, for example, although the embryo presented in Fig. 4C developed to multicellular stage, it had less-flattened pre-compacted appearance of outer cells and although initiated, could not maintain cavity formation. It is also noteworthy that similar defects in compaction were observed in 60% (*n* = 10) of the time-lapse filmed embryos injected with *Cdx2*-specific siRNA (Supplementary Movie 4). Interestingly, compaction was often delayed until after entry into the 16-cell stage and outer cells retained a rounded appearance (Supplementary Movie 6).

In the molecular characterisation of the defects observed after depletion of both maternal and zygotic pool of *Cdx2*, we also assessed its impact on the expression of trophectoderm-related genes. We found that the immuno-staining observed at the membranes of all trophectoderm cells in control blastocysts with Troma1 antibody was virtually absent from *Cdx2* RNAi embryos cultured to the equivalent stage (Fig. 4D). Further characterisation of trophectoderm-specific genes at the mRNA level verified the *Cdx2* depletion and showed that *Gata3* levels were reduced by 44% and *Eomes* mRNA was undetectable by the 16-cell stage, in contrast to control embryos (Figs. 4B and B′). The effect on *Eomes* expression was also confirmed on the protein level in *Cdx2*-depleted embryos at stages equivalent to blastocyst (Supplementary Fig. 4). Thus, the compound reductions in *Gata3*, Troma1 and *Eomes* expression indicate that Cdx2 depletion from the zygote stage affects trophectoderm specification. Interestingly, Cdx2 depletion resulted in an over 3-fold increase in the levels of *Tead4* mRNA (Fig. 4B), a transcription factor thought to act upstream of Cdx2 from studies on zygotic gene knockout models (Nishioka et al., 2009; Nishioka et al., 2008; Yagi et al., 2007), possibly indicating a regulative mechanism of the embryo in response to *Cdx2* depletion.

Additionally, we assayed the expression levels of key pluripotency-related factors. We found that while we could detect little effect on *Oct4* transcripts, after the *Cdx2* depletion we could no longer observe *Nanog* mRNA expression by the 16-cell stage (Figs. 4B, B′). The lack of effect on *Oct4* mRNA might reflect the high levels of maternally inherited transcript known to exist whereas the absence of *Nanog* transcripts at the 16-cell stage suggest that not only cell polarity or trophectoderm-related genes are affected upon depletion of early pool of *Cdx2* mRNA. Thus, the early functional roles of Cdx2 are likely to be more wide ranging than previously anticipated from zygotic gene knockout studies. Taken together, these results indicate the importance of interplay between cell polarity and Cdx2 expression and affirm the importance of Cdx2 function from the early stages of development to ultimately specify outside versus inside cells defined by the blastocyst stage by a functional trophectoderm.

### Depletion of maternal pool of Cdx2 by antisense morpholinos confirms the role of maternal pool of *Cdx2 mRNA*

Since antisense morpholino oligonucleotides designed to target gene-specific 5′UTR or translational start sites have been recently demonstrated to mediate highly specific gene knockdown in mouse zygotes (Foygel et al., 2008), we also applied this approach to knockdown *Cdx2* expression in the early zygote. This allowed us to compare the observed phenotype with those seen after injection of *Cdx2*-specific dsRNA and siRNA at the same stage.

**Fig. 5:**
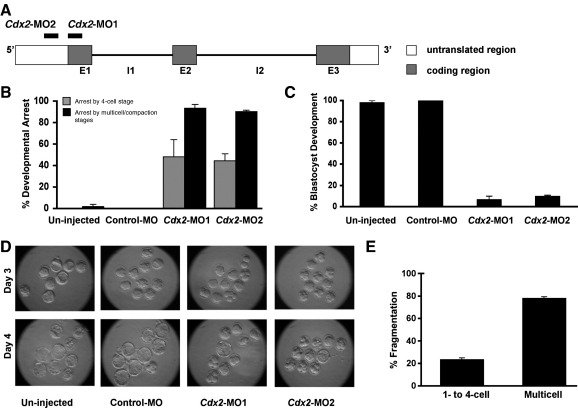
Cdx2 depletion from the zygote stage by antisense morpholino arrests pre-implantation development. (A) Non-overlapping sequences that are targeted by the two morpholinos used in this study – *Cdx2*-MO1 and *Cdx2*-MO2 – at the *Cdx2* locus. (B) *Cdx2*-MO1- and *Cdx2*-MO2-injected embryos arrested by the 4-cell stage (grey bars) and by the multicell/compaction stages (black bars), while uninjected embryos and control morpholino (Control-MO)-injected embryos reached blastocyst stage. (C) Only a small percentage of embryos injected with *Cdx2*-MOs developed to the blastocyst stage, compared to near-100% blastocyst development in controls. (D) Representative images observed for each condition at days 3 and 4 of *in vitro* culture after injection. (E) Embryos injected with *Cdx2* morpholinos that were also arrested, showed high fragmentation rates.

We designed two morpholinos, *Cdx2*-MO1 and *Cdx2*-MO2, that specifically targeted non-overlapping sequences at the translational start site and in the 5′UTR respectively, of mouse *Cdx2* transcript (Fig. 5A). We found that following their microinjection into zygotes, the rate of developmental arrest was dramatically higher than those observed for uninjected embryos and those injected with control morpholino (Control-MO) (Figs. 5B, C). Embryos injected with *Cdx2*-MO1 and *Cdx2*-MO2 showed similar rates of developmental arrest at 93.3 ± 3.3% and 90.3 ± 1.1% by the multicell/compaction stages. These rates of developmental arrest were significantly higher than those of controls (*p* < 0.005; Figs. 5B, C). Consequently, only 6.7 ± 3.3% and 9.7 ± 1.2% of the *Cdx2*-MO1- and *Cdx2*-MO2-injected embryos, respectively, developed into blastocysts, compared to nearly 100% of control embryos (*p* < 0.005; Figs. 5B–D).

Consistent with irregular cell divisions and cell death observed in Cdx2 RNAi embryos and in contrast to morpholino-mediated knockdown of *Oct4* and *Ccna2* (Foygel et al., 2008), *Cdx2* knockdown using either of two morpholinos also resulted in fragmentation of embryos arrested by multicell/compaction stages. Specifically, the *Cdx2* morpholinos resulted in 77.9 ± 1.5% of embryos fragmenting by the multicell/compaction stages (Fig. 5E). We also found that some of the *Cdx2* knockdown embryos that compacted subsequently reverted to a pre-compaction appearance about 24 hours later.

Thus, antisense morpholino oligonucleotides directed against *Cdx2* from the zygote stage resulted in a similar phenotype to Cdx2 depletion by RNAi and both strategies resulted in much earlier phenotypes than those observed after elimination of only zygotic *Cdx2.* Taken together, these data also indicate the requirement for Cdx2 prior to the appearance of morphologically apparent trophectoderm cells and indicate that Cdx2 is essential for development through cell polarisation/compaction up until the blastocyst stages.

## Discussion

The expression status of the *Cdx2* gene in the earliest stages of mouse embryo development has been a subject of debate (Roberts et al., 2007). Notwithstanding this, we present both new and existing data (Wang et al., 2004) that clearly indicates the presence of *Cdx2* mRNA in the mouse egg. Furthermore, we find that this maternal pool is functionally drawn upon during the earliest stages of pre-implantation development. We reach this conclusion because we observed an earlier and more severe phenotype after inhibiting *Cdx2* expression starting from the zygote stage using three highly specific gene knockdown approaches – injection of either dsRNA or siRNA or antisense morpholinos that target the *Cdx2* transcript – compared to the trophectoderm maintenance/late blastocyst phenotype previously reported for deletion of only the zygotic copies of the *Cdx2* gene (Ralston and Rossant, 2008; Strumpf et al., 2005). Specifically, upon Cdx2 depletion by either RNAi or morpholinos, the embryos arrest before the blastocyst stage, showing defects in cell polarisation and compaction, their developmental progression is much slower with cell cycle lengths significantly elongated, gene expression associated with trophectoderm or pluripotency-related factors alters, they show increased cell death and, finally, more severe defects in the establishment and maintenance of the blastocyst cavity. These phenotypic effects resonate with the recent finding that development of Rhesus monkey zygotes injected with *Cdx2*-specific antisense morpholinos is also compromised with the first arrests becoming evident at the 8-cell stage, coincident with the timing of compaction and polarisation, and that under half the embryos ever reach the early blastocyst stage (Sritanaudomchai et al., 2009). That the effects we observe here are specific to Cdx2 depletion is supported by the fact we obtain similar early phenotypes using three independent knockdown approaches, albeit the morpholino approach seems to act slightly faster, possibly reflecting a more direct mechanism of action when compared to dsRNA processing. Furthermore, the characteristic developmental phenotype associated with Cdx2 depletion can be ‘rescued’ by over-expression of synthetic *Cdx2* mRNA. The effects we observe with the RNAi-based strategy cannot be due to inherent toxicity of injected dsRNA or siRNA given that our control dsRNA and siRNA, injected at the same concentration as the *Cdx2*-specific constructs, did not adversely effect development. Moreover, when we used the same construct and injection conditions to deplete Cdx2 at later developmental stage, effectively from the 4- to 8-cell stage in half the embryo, the effective doubling in concentration of dsRNA accounted for by injecting the smaller cytoplasmic volume of a 2-cell blastomere versus a zygote did not adversely effect development *per se* (Jedrusik et al., 2008). However, this intervention drove the allocation of injected cell progeny with reduced Cdx2 levels to occupy the pluripotent ICM rather than trophectoderm of morphologically normal blastocysts.

The observed differences in phenotype between the *Cdx2*^*−*^^*/*^^*−*^ embryos and the *Cdx2* knockdown models presented here, can be explained by a maternal effect of *Cdx2* mRNA. Deletion of zygotic *Cdx2* alone (Ralston and Rossant, 2008; Strumpf et al., 2005) would not abrogate the initial functional roles, *e.g.* cell polarisation, compaction or trophectoderm specification, because the maternal pool of *Cdx2* mRNA provided by the egg cytoplasm would be sufficient to sustain the embryo through these early stages and to initiate trophectoderm cell fate. Therefore, zygotic deletion of *Cdx2* results in the observed milder phenotype, as maintenance of trophectoderm function, unlike trophectoderm specification, likely relies on zygotically derived *Cdx2* expression. Indeed our data indicate that from the 16-cell stage, a large component of the Cdx2 protein is zygotically derived (Fig. 1E). In contrast, all three knockdown approaches described here would not only abrogate zygotic *Cdx2* mRNA function but also that of its maternal counterpart. This simultaneous loss of maternal and zygotic *Cdx2* function would in turn result in the herein observed early phenotypes associated with the loss of trophectoderm specification that precede the establishment of inner and outer cells. It is worth noting that the potential importance of a maternal pool of *Cdx2* mRNA had been eluded to, although not demonstrated, by the previous studies. Specifically, it was demonstrated that the RNAi-mediated depletion of *Cdx2* from the 4- to 8-cell stage had a greater effect on cell polarity and allocation (Jedrusik et al., 2008) than the knockout of the zygotic *Cdx2* (Ralston and Rossant, 2008; Strumpf et al., 2005). From our current perspective, this stronger phenotype could be explained by a maternal effect of *Cdx2* mRNA. Furthermore it suggests that by the 8-cell stage at least some of this is translated to yield Cdx2 protein, as the observed phenotype after RNAi taking place at the 4-cell stage was not as severe as when *Cdx2* mRNA is eliminated from the zygote stage. This is further supported by the existence of clearly detectable Cdx2 protein in 16-cell embryo nuclei in which global transcription has been blocked from the 4- to 8-cell stage transition.

During normal development, Cdx2 protein expression is heterogeneous at the 8-cell stage with on average nuclei of only 2 cells per embryo exhibiting robust positive staining (Jedrusik et al., 2008). This Cdx2 distribution is not just heterogeneous but also asymmetrical in a manner dependent upon the orientation and order of cleavages of the 2-cell blastomeres that generated them (Jedrusik et al., 2008). Such cells with the highest *Cdx2* levels make a biased contribution to trophectoderm (Bischoff et al., 2008); are the least pluripotent (Piotrowska-Nitsche et al., 2005); and have the lowest levels of specific histone H3 arginine methylation, an epigenetic mark known to correlate with pluripotency (Torres-Padilla et al., 2007; Wu et al., 2009). The asymmetry of Cdx2 protein at the 8-cell stage leads to the question of whether the maternal *Cdx2* mRNA we now detect is in any way asymmetrically distributed, either initially in the egg or subsequently in the early cleavage stages. Our mRNA FISH results do not support a drastic localisation/partitioning of *Cdx2* mRNA at the zygote stage; equally it does not exclude the possibility of subtle asymmetries in inheritance that could then be subsequently amplified.

Recent studies have described the function of other essential trophectoderm transcription factors, Tead4 (Nishioka et al., 2008; Yagi et al., 2007) and Gata3 (Home et al., 2009), reported to act upstream of *Cdx2* expression. *Tead4*^*−*^^*/*^^*−*^ embryos display pre-implantation lethality and do not initiate cavitation or zygotic *Cdx2* expression (Nishioka et al., 2008; Yagi et al., 2007). The developmental timing of the *Tead4*^*−*^^*/*^^*−*^ phenotype together with the reported onset of *Tead4* expression after zygotic genome activation suggests that its loss of function would not affect the earliest/maternal levels of either *Cdx2* mRNA or protein, indicated by this study. Indeed, there is evidence for low Cdx2 protein levels in a subset of *Tead4*^*−*^^*/*^^*−*^ blastomeres by the morula stage (Nishioka et al., 2008). It is plausible that any Cdx2 protein in Tead4^*−*^^/^^*−*^ embryos could be provided from the maternal pool of *Cdx2* mRNA described here. Thus, it cannot yet be ruled out that Tead4 is upstream of zygotically derived Cdx2 but that maternally provided Cdx2 exists independently of Tead4. In fact the elimination of maternal Cdx2 leads to an increase in *Tead4* transcript levels as we show here. This would be consistent with the role of Tead4 in ensuring *Cdx2* is correctly expressed in the outer but not the inner cells of the embryo once they have been derived (Nishioka et al., 2009). In the case of *Gata3* depletion using RNAi, *Cdx2* mRNA expression was shown to be reduced by around 55% in embryos that exhibit a marked arrest around the morula/blastocyst transition (Home et al., 2009). This comparatively later phenotype is somewhat between that reported here and that when the zygotic *Cdx2* is removed (Ralston and Rossant, 2008; Strumpf et al., 2005) and suggests Gata3 acts to influence *Cdx2* expression at the zygotic level rather than affecting maternally derived Cdx2. Indeed the fact that we observe a 44% reduction in *Gata3* expression following Cdx2 depletion from the zygote stage suggests the existence of a mutually reinforcing feedback loop, operating on the level of zygotic transcription that is independent of maternally derived Cdx2. However, if maternally provided Cdx2 is removed, this loop is compromised by a reduced capacity for *Gata3* activation.

It is known that *Cdx2* transcript levels increase as the inner and outer cell populations begin to be established (Wang et al., 2004). Thus, the earliest effect of Tead4 function on *Cdx2* expression appears to regulate Cdx2 levels in response to cell position from the 8-cell stage onwards. This relatively late and developmental stage-specific function of Tead4 in *Cdx2* regulation would also explain why *Tead4*^*−*^^*/*^^*−*^ 8-cell blastomeres polarise (Nishioka et al., 2008) despite our finding that perturbations in *Cdx2* expression prior to the 8-cell stage alters the degree of polarisation and the expression of polarisation-related genes. In agreement with this, recent evidence suggests existence of a mutually reinforcing relationship between Cdx2 and cell polarity which determine cell fate/position (Jedrusik et al., 2008; Plusa et al., 2005). Thus, the cell polarisation required to initiate the first cell fate decision may be mediated in part by maternally derived Cdx2 and subsequently maintained by a Cdx2 executed program directed in the correct population of outer cells by Tead4.

In light of the results we present here, we would like to reconsider how Cdx2 mediates the specification of trophectoderm cell fate. Our results provide evidence that low but functionally significant levels of maternally derived *Cdx2* mRNA are translated in the early cleavage stage embryos, and if this is prevented by *Cdx2* RNAi or antisense morpholinos, the phenotypic effect is more severe than targeting the zygotic *Cdx2* loci alone, resulting in defective cell polarisation at the 8- to 16-cell stages. Because Cdx2 protein positively auto-regulates its own expression (Saegusa et al., 2007), the accumulation of maternally derived Cdx2 protein could prime zygotic *Cdx2* expression. Once a critical threshold of Cdx2 protein is reached at the 8- to 16-cell transition, zygotic *Cdx2* transcription then robustly ensues, resulting in the large increases in the levels of *Cdx2* transcripts observed around this time (Wang et al., 2004). This would also promote/maintain blastomere polarisation and ultimately trophectoderm integrity and function by the blastocyst stage. Differential levels of Cdx2 protein expression reported in number of studies among blastomeres at the 8-cell stage (Jedrusik et al., 2008; Ralston and Rossant, 2008) could be explained by even a very small bias in the inheritance of the maternal *Cdx2* mRNA between blastomeres given the positive feedback loop mechanism. Equally, differences among blastomeres may be also due to differential transcriptional activation. Even small differences would have the opportunity to be greatly amplified during the long cell cycles of the first three cleavage divisions. Notwithstanding this, any larger bias in maternal *Cdx2* mRNA inheritance, akin to that suggested by the fact that approximately 40% of embryos have substantially higher levels of *Cdx2* transcript in the cells derived from vegetal blastomeres (Jedrusik et al., 2008), would result in still greater asymmetries by the 8-cell stage.

The differential *Cdx2* phenotypes between the zygotic knockout model and the simultaneous knockdown of maternal and zygotic transcripts are reminiscent of the recently reported earlier role for Oct4 that was unmasked by morpholino-mediated knockdown of *Oct4* (Foygel et al., 2008). In the mouse knockout model, *Oct4* was known for its requirement in ICM expansion and pluripotency (Nichols et al., 1998), as *Cdx2* was known for trophectoderm maintenance; both genes were known for their critical role in development after formation of the early blastocyst. However, neither gene was suspected to be required for blastocyst formation. In the case of *Oct4*, it was surprising that blocking gene function from the zygote stage did not result in the anticipated induction of *Cdx2* or reduction in *Sox2* expression that would be predicted from the zygotic gene knockout models (Foygel et al., 2008). Similarly, we find that inhibiting *Cdx2* expression in this study fails to induce *Nanog* expression and that its levels are undetectable by the 16-cell stage. Hence, it appears that the earlier roles of Cdx2 could be quite distinct from those characterized at later stages, as has been described for Oct4. Indeed our finding that *Cdx2* has an early role raises the question that other genes that have maternal and zygotic contribution may also function earlier in development than previously anticipated. Using gene knockdown strategies, the study of maternal effect would not only be restricted to oocyte-specific genes, such as *Zar1* and *Nobox1*, that are not transcribed after zygotic genome activation (Tong et al., 2000; Wu et al., 2003). It is possible that a broad application of this alternative paradigm would provide access to understand the essential and early steps for lineage specification in the early embryo.

## Appendix A Supplementary data

Fig. 1 Immuno-fluorescence staining specific for Cdx2 protein in representative zygotes, 2-cell, 4-cell and 8-cell stage embryos. DAPI DNA counter-stain and DIC images are shown for reference; scale bar 10 μm. Note the extent of clear nuclear accumulation of Cdx2 protein is heterogeneous and become evident only in a sub-population of 8-cell blastomere nuclei. A negative control 2-cell stage embryo stained with only secondary antibody is shown for reference.

Fig. 2 Q-RT-PCR assay for α-amanitin sensitive (§—*Eif1a*) and α-amanitin insensitive (***†***—*Mdm4*) gene transcripts from late 2-cell embryos pretreated with (red bars) or without (blue bars) α-amanitin (24 µg/ml) from the early zygote stage (α-amanitin sensitivity personally communicated by Richard Schultz). Gene expression levels are normalized to the level of *ActB* and error bars represent SEM from experimental triplicates. Note the complete block in the transcriptional activation of the *Eif1a* gene versus *Mdm4* in the presence of α-amanitin that normally occurs during zygotic genome activation, confirming its efficacy in our experimental system. Also note that *Mdm4* levels appear to increase with α-amanitin treatment versus without but that this is due to falls in the levels of *ActB* transcripts that are not insensitive to α-amanitin treatment.

Fig. 3 Injection of zygotes with siRNA oligos specific for *Cdx2* mRNA phenocopies the effect of injecting long dsRNA designed to target *Cdx2*. (A) Early zygotes were microinjected with either *Cdx2*-specific siRNA (plus *DsRed* mRNA to confirm injection—upper panels), *DsRed* mRNA alone (middle panels) or a control siRNA (without any homology in the mouse genome and of medium GC nucleotide content, plus *DsRed*—lower panels) and cultured *in vitro* until the late blastocyst stage. At indicated time points, embryos were manually inspected and the developmental stage attained morphologically judged. Representative images are shown. Note lack of fully expanded/hatching blastocysts in *Cdx2* siRNA injected embryos versus control conditions at E4.5 and arrest at 8- to 16-cell stage associated with cell death. **(**B) Embryos fixed at the blastocyst stage (relative to controls) that developed from zygotes injected with *Cdx2* siRNA immuno-stained for Cdx2. Upper panel shows a control embryo with Cdx2 positive cell nuclei in the trophectoderm. The lower three panels show representative embryos from the *Cdx2* siRNA injected group. The first row is representative of embryos that arrest at multicelluar morula stages and have undetectable levels of Cdx2 protein, akin to group 1 embryos in the dsRNA assays (Fig. 2). The middle row represents embryos that attempted to form ‘blastocyst-like’ structures but have fewer Cdx2 positive cells and lower expression levels (more similar to group 2 embryos in the dsRNA assays—Fig. 2). The lowest panel shows an example of some embryos that do not down-regulate Cdx2 efficiently after *Cdx2*-specific siRNA injection. DNA stains (Hoechst) and DIC images are shown; scale bar 10 µM. (C) Embryos cultured from zygotes injected with *Cdx2* siRNA express lower levels of, and mislocalise, aPKC protein at the 8- to 16-cell stage. Upper panels show an example of a siRNA control embryo with apical aPKC protein expression and the lower two panels with lower expression and not restricted to the apical pole. DNA stains (Hoechst) and DIC images are shown; scale bar 10 µM.

Fig. 4 Eomesodermin (Eomes—green) and Cdx2 (red) co-immuno-fluorescence staining at a stage equivalent to blastocyst, in either representative control embryos or those co-injected with long dsRNA specific for *Cdx2* (*Cdx2* dsRNA). DNA Hoeschst nuclear stains (blue) and DIC images are shown for reference, scale bar 10 µM.

Table 1 Proportional contribution to inside and outside compartment of live and dying cells in the second group (developing past 16-cell stage) in Cdx2-depleted embryos (upper panel—see text for details). Proportional contribution to inside and outside compartment of live and dying cells in control embryos that were injected with *DsRed* mRNA only (lower panel).

Movies 1–3 Time-lapse movies of embryos depleted of Cdx2 at the zygote stage from Figs. 2D–F, respectively. Three-dimensional reconstruction of spatial localisation of blastomeres centres (right panel); DIC movie of the same embryo (left panel). Centres of the nuclei of individual cells are marked either red (live cells) or white (cells that just died or are about to die).

Movie 4 Time-lapse movie of embryos injected with Cdx2-specific siRNA at the zygotes stage. Three-dimensional reconstruction of spatial localisation of blastomeres centres (right panel); DIC movie of the same embryo (left panel). Centres of the nuclei of individual cells are marked either red (live cells) or white (cells that just died or are about to die). Note 70% (7/10) of the time-lapsed filmed embryos arrest at the 8- to 16-cell stages.

Movie 5 Time-lapse movie of an embryo injected with *Cdx2*-specific dsRNA and rescuing *Cdx2* mRNA at the zygote stage detailing the Cdx2 specificity of the observed phenotype. Three-dimensional reconstruction of spatial localisation of blastomeres centres (right panel); DIC movie of the same embryo (left panel). Centres of the nuclei of individual cells are marked either red (live cells) or white (cells that just died or are about to die). Note that embryo is able to form and maintain blastocoel.

Movie 6 Time-lapse movie of an embryo injected with *Cdx2*-specific siRNA at the zygote stage with defective and delayed compaction. Three-dimensional reconstruction of spatial localisation of blastomeres centres (right panel); DIC movie of the same embryo (left panel). Centres of the nuclei of individual cells are marked either red (live cells) or white (cells that just died or are about to die). Note atypical rounded appearance of blastomeres after entry into 16-cell stage in DIC image.

## References

[bib1] Bischoff M., Parfitt D.E., Zernicka-Goetz M. (2008). Formation of the embryonic-abembryonic axis of the mouse blastocyst: relationships between orientation of early cleavage divisions and pattern of symmetric/asymmetric divisions. Development.

[bib2] Chazaud C., Yamanaka Y., Pawson T., Rossant J. (2006). Early lineage segregation between epiblast and primitive endoderm in mouse blastocysts through the Grb2-MAPK pathway. Dev. Cell.

[bib3] Copp A.J. (1978). Interaction between inner cell mass and trophectoderm of the mouse blastocyst. I. A study of cellular proliferation. J. Embryol. Exp. Morphol..

[bib4] Deb K., Sivaguru M., Yong H.Y., Roberts R.M. (2006). Cdx2 gene expression and trophectoderm lineage specification in mouse embryos. Science.

[bib5] Foygel K., Choi B., Jun S., Leong D.E., Lee A., Wong C.C., Zuo E., Eckart M., Reijo Pera R.A., Wong W.H., Yao M.W. (2008). A novel and critical role for Oct4 as a regulator of the maternal-embryonic transition. PLoS ONE.

[bib6] Graham C.F., Deussen Z.A. (1978). Features of cell lineage in preimplantation mouse development. J. Embryol. Exp. Morphol..

[bib7] Hadjantonakis A.K., Papaioannou V.E. (2004). Dynamic in vivo imaging and cell tracking using a histone fluorescent protein fusion in mice. BMC Biotechnol..

[bib8] Home P., Ray S., Dutta D., Bronshteyn I., Larson M., Paul S. (2009). GATA3 is selectively expressed in the trophectoderm of peri-implantation embryo and directly regulates Cdx2 gene expression. J. Biol. Chem..

[bib9] Jedrusik A., Parfitt D.E., Guo G., Skamagki M., Grabarek J.B., Johnson M.H., Robson P., Zernicka-Goetz M. (2008). Role of Cdx2 and cell polarity in cell allocation and specification of trophectoderm and inner cell mass in the mouse embryo. Genes Dev..

[bib10] Johnson M.H., Ziomek C.A. (1982). Cell subpopulations in the late morula and early blastocyst of the mouse. Dev. Biol..

[bib11] Livak K.J., Schmittgen T.D. (2001). Analysis of relative gene expression data using real-time quantitative PCR and the 2(-Delta Delta C(T)) Method. Methods.

[bib12] Nichols J., Zevnik B., Anastassiadis K., Niwa H., Klewe-Nebenius D., Chambers I., Scholer H., Smith A. (1998). Formation of pluripotent stem cells in the mammalian embryo depends on the POU transcription factor Oct4. Cell.

[bib13] Nishioka N., Inoue K., Adachi K., Kiyonari H., Ota M., Ralston A., Yabuta N., Hirahara S., Stephenson R.O., Ogonuki N., Makita R., Kurihara H., Morin-Kensicki E.M., Nojima H., Rossant J., Nakao K., Niwa H., Sasaki H. (2009). The Hippo signaling pathway components Lats and Yap pattern Tead4 activity to distinguish mouse trophectoderm from inner cell mass. Dev. Cell.

[bib14] Nishioka N., Yamamoto S., Kiyonari H., Sato H., Sawada A., Ota M., Nakao K., Sasaki H. (2008). Tead4 is required for specification of trophectoderm in pre-implantation mouse embryos. Mech. Dev..

[bib15] Niwa H., Toyooka Y., Shimosato D., Strumpf D., Takahashi K., Yagi R., Rossant J. (2005). Interaction between Oct3/4 and Cdx2 determines trophectoderm differentiation. Cell.

[bib16] Pedersen R.A., Wu K., Balakier H. (1986). Origin of the inner cell mass in mouse embryos: cell lineage analysis by microinjection. Dev. Biol..

[bib17] Piotrowska-Nitsche K., Perea-Gomez A., Haraguchi S., Zernicka-Goetz M. (2005). Four-cell stage mouse blastomeres have different developmental properties. Development.

[bib18] Plusa B., Frankenberg S., Chalmers A., Hadjantonakis A.K., Moore C.A., Papalopulu N., Papaioannou V.E., Glover D.M., Zernicka-Goetz M. (2005). Downregulation of Par3 and aPKC function directs cells towards the ICM in the preimplantation mouse embryo. J. Cell Sci..

[bib19] Ralston A., Rossant J. (2008). Cdx2 acts downstream of cell polarization to cell-autonomously promote trophectoderm fate in the early mouse embryo. Dev. Biol..

[bib20] Roberts R.M., Sivaguru M., Yong H.Y. (2007). Retraction. Science.

[bib21] Saegusa M., Hashimura M., Kuwata T., Hamano M., Wani Y., Okayasu I. (2007). A functional role of Cdx2 in beta-catenin signaling during transdifferentiation in endometrial carcinomas. Carcinogenesis.

[bib22] Schnabel R., Hutter H., Moerman D., Schnabel H. (1997). Assessing normal embryogenesis in Caenorhabditis elegans using a 4D microscope: variability of development and regional specification. Dev. Biol..

[bib23] Sritanaudomchai H., Sparman M., Tachibana M., Clepper L., Woodward J., Gokhale S., Wolf D., Hennebold J., Hurlbut W., Grompe M., Mitalipov S. (2009). CDX2 in the formation of the trophectoderm lineage in primate embryos. Dev. Biol..

[bib24] Strumpf D., Mao C.A., Yamanaka Y., Ralston A., Chawengsaksophak K., Beck F., Rossant J. (2005). Cdx2 is required for correct cell fate specification and differentiation of trophectoderm in the mouse blastocyst. Development.

[bib25] Svoboda P., Stein P., Hayashi H., Schultz R.M. (2000). Selective reduction of dormant maternal mRNAs in mouse oocytes by RNA interference. Development.

[bib26] Thomas F.C., Sheth B., Eckert J.J., Bazzoni G., Dejana E., Fleming T.P. (2004). Contribution of JAM-1 to epithelial differentiation and tight-junction biogenesis in the mouse preimplantation embryo. J. Cell Sci..

[bib27] Tong Z.B., Gold L., Pfeifer K.E., Dorward H., Lee E., Bondy C.A., Dean J., Nelson L.M. (2000). Mater, a maternal effect gene required for early embryonic development in mice. Nat. Genet..

[bib28] Torres-Padilla M.E., Parfitt D.E., Kouzarides T., Zernicka-Goetz M. (2007). Histone arginine methylation regulates pluripotency in the early mouse embryo. Nature.

[bib29] Wang Q.T., Piotrowska K., Ciemerych M.A., Milenkovic L., Scott M.P., Davis R.W., Zernicka-Goetz M. (2004). A genome-wide study of gene activity reveals developmental signaling pathways in the preimplantation mouse embryo. Dev. Cell.

[bib30] Wianny F., Zernicka-Goetz M. (2000). Specific interference with gene function by double-stranded RNA in early mouse development. Nat. Cell Biol..

[bib31] Wu Q., Bruce A.W., Jedrusik A., Ellis P.D., Andrews R.M., Langford C.F., Glover D.M., Zernicka-Goetz M. (2009). CARM1 is Required in ES Cells to Maintain Pluripotency and Resist Differentiation. Stem Cells.

[bib32] Wu X., Viveiros M.M., Eppig J.J., Bai Y., Fitzpatrick S.L., Matzuk M.M. (2003). Zygote arrest 1 (Zar1) is a novel maternal-effect gene critical for the oocyte-to-embryo transition. Nat. Genet..

[bib33] Yagi R., Kohn M.J., Karavanova I., Kaneko K.J., Vullhorst D., DePamphilis M.L., Buonanno A. (2007). Transcription factor TEAD4 specifies the trophectoderm lineage at the beginning of mammalian development. Development.

