# Supplementary material for: Maternally and zygotically provided Cdx2 have novel and critical roles for early development of the mouse embryo
Source: Dev Biol. 2010 Aug 1;344(1-2):66–78. doi: 10.1016/j.ydbio.2010.04.017 (PMC2954319; doi:10.1016/j.ydbio.2010.04.017)
Supplement: Fig. 1 — Immuno-fluorescence staining specific for Cdx2 protein in representative zygotes, 2-cell, 4-cell and 8-cell stage embryos. DAPI DNA counter-stain and DIC images are shown for reference; scale bar 10 μm. Note the extent of clear nuclear accumulation of Cdx2 protein is heterogeneous and become evident only in a sub-population of 8-cell blastomere nuclei. A negative control 2-cell stage embryo stained with only secondary antibody is shown for reference. [file mmc7.pdf]

Supplementary Figure 1

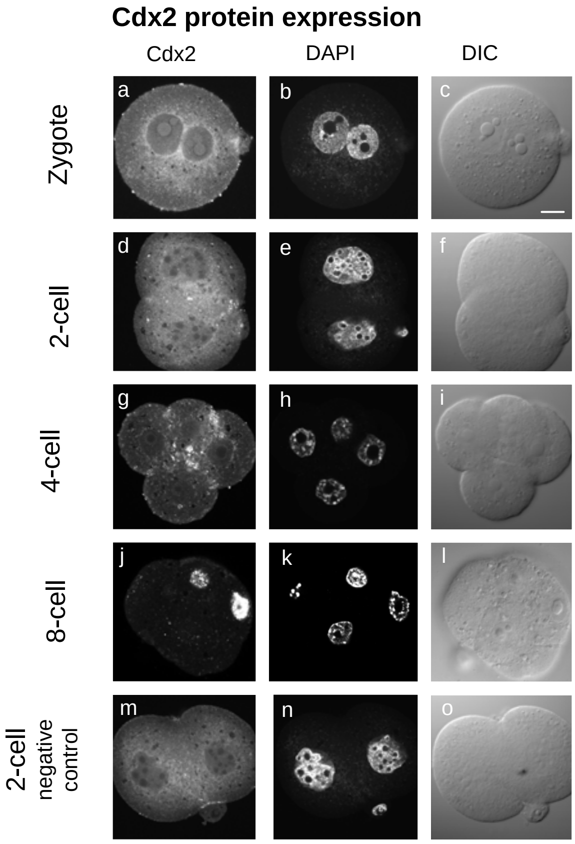

Supplementary Figure 2

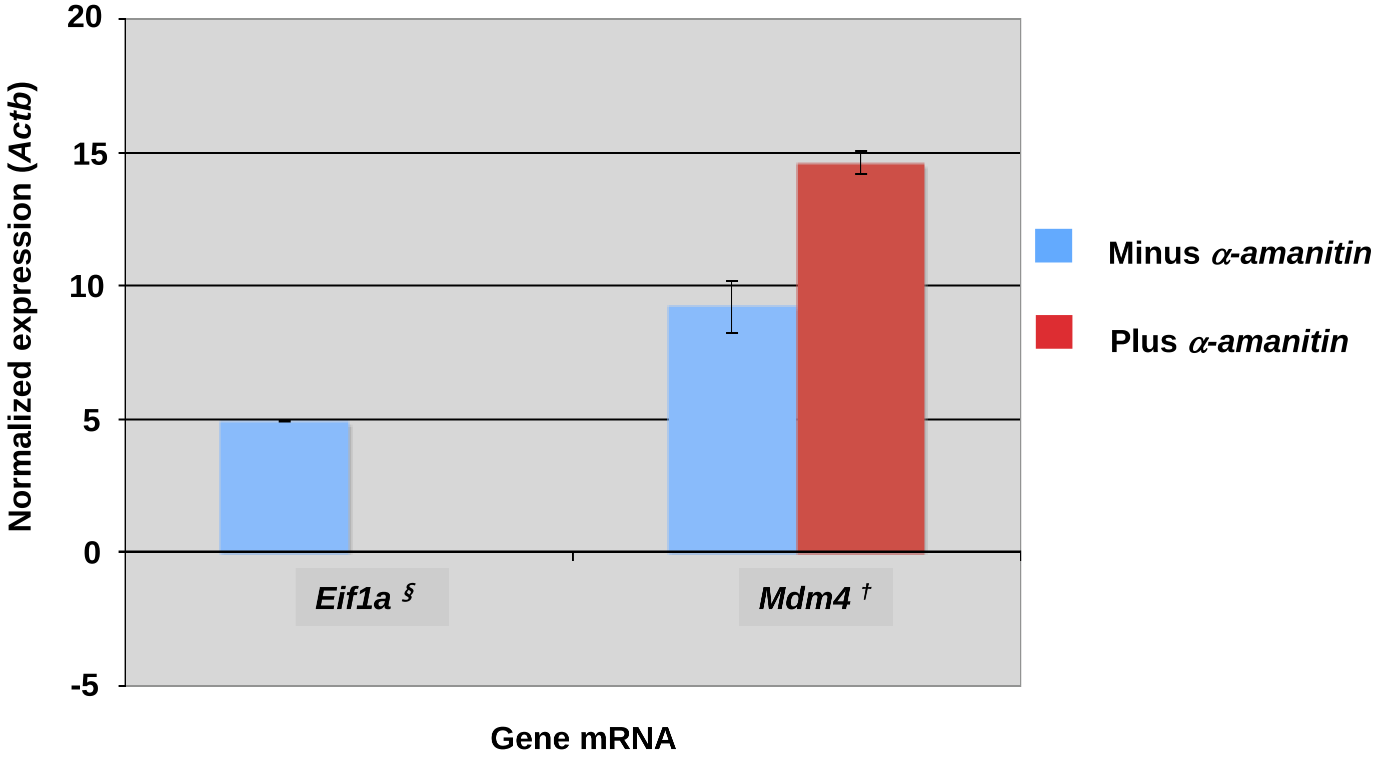

§ α-amanitin sensitive transcript
† α-amanitin insensitive transcript

Supplementary Figure 3

A Developmental progression of siRNA treated embryos

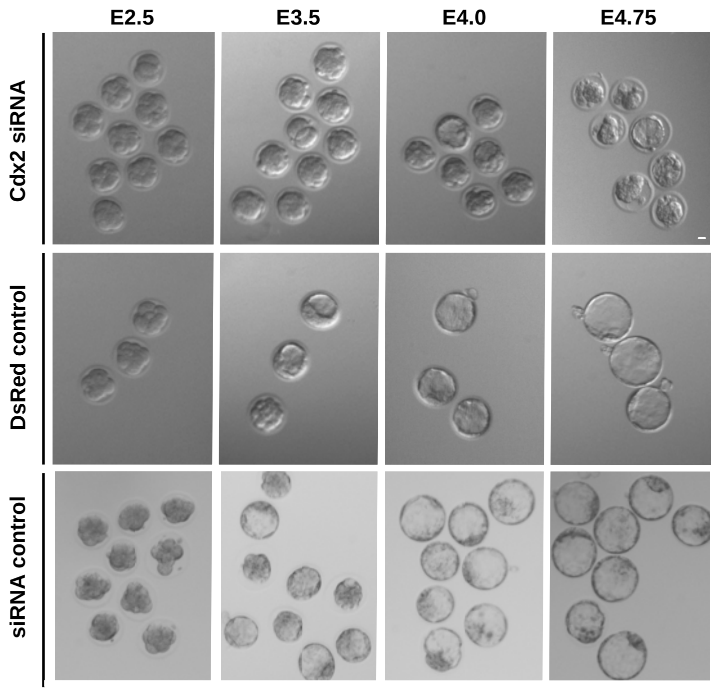

B Cdx2 expression in Cdx2 siRNA embryos

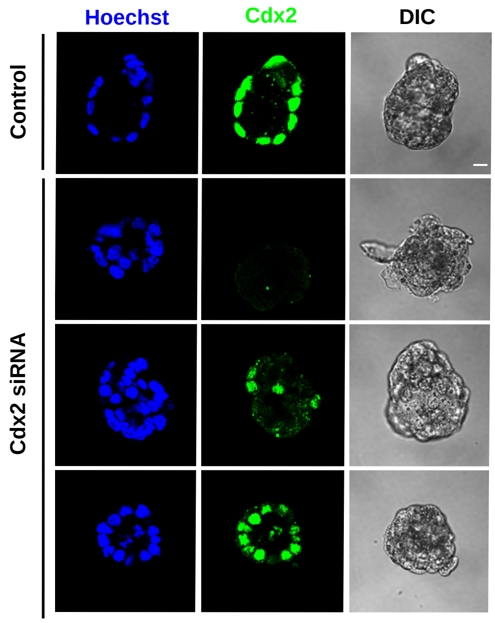

C Polarity in Cdx2 siRNA embryos

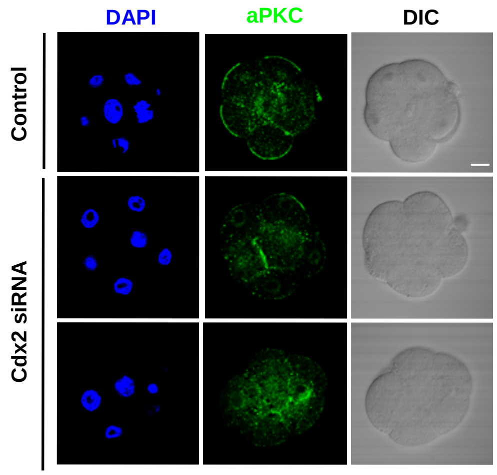

Supplementary Figure 4

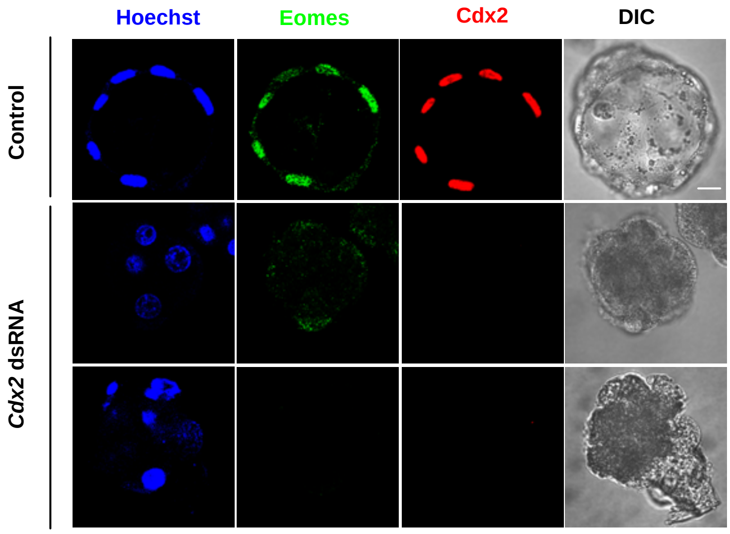

Supplementary Table 1

Cell allocation in Cdx2 RNAi embryos

No	Number of cells			Inside position			Outside position		
	Total	Live	Dead	Total	Live	Dead	Total	Live	Dead
1	24	18	6	11	8	3	13	10	3
2	29	29	0	10	10	0	19	19	0
3	30	18	12	13	10	3	17	8	9
4	30	30	0	14	14	0	16	16	0
5	32	25	7	17	12	5	15	13	2
6	32	27	5	11	11	0	21	21	0
7	32	32	0	14	12	2	18	15	3
8	32	32	0	6	6	0	26	26	0
9	35	34	1	18	17	1	17	17	0
**Av**	**30.7**	**27.2**	**3.4**	**12.7**	**11.1**	**1.6**	**18.0**	**16.1**	**1.9**

Cell allocation in control embryos

No	Number of cells			Inside position			Outside position		
	Total	Live	Dead	Total	Live	Dead	Total	Live	Dead
1	32	32	0	11	11	0	21	21	0
2	32	32	0	9	9	0	23	23	0
3	32	32	0	10	10	0	22	22	0
4	32	32	0	10	10	0	22	22	0
5	32	32	0	11	11	0	21	21	0
6	32	32	0	10	10	0	22	22	0
7	33	33	0	12	12	0	21	21	0
**Av**	**32.1**	**32.1**	**0**	**10.4**	**10.4**	**0**	**21.7**	**21.7**	**0**
